# Exploring the Importance of Differential Expression of Autophagy Markers in Term Placentas from Late-Onset Preeclamptic Pregnancies

**DOI:** 10.3390/ijms25042029

**Published:** 2024-02-07

**Authors:** Luis M. Garcia-Puente, Cielo García-Montero, Oscar Fraile-Martinez, Julia Bujan, Juan A. De León-Luis, Coral Bravo, Patrocinio Rodríguez-Benitez, Laura López-González, Raul Díaz-Pedrero, Melchor Álvarez-Mon, Natalio García-Honduvilla, Miguel A. Saez, Miguel A. Ortega

**Affiliations:** 1 Department of Medicine and Medical Specialities, Faculty of Medicine and Health Sciences, University of Alcalá, 28801 Alcalá de Henares, Spain; lmgarciapuente@gmail.com (L.M.G.-P.); cielo.gmontero@gmail.com (C.G.-M.); oscarfra.7@hotmail.com (O.F.-M.); mjulia.bujan@uah.es (J.B.); melchor.alvarezdemon@uah.es (M.Á.-M.); natalio.garcia@uah.es (N.G.-H.); msaega1@oc.mde.es (M.A.S.); 2 Ramón y Cajal Institute of Sanitary Research (IRYCIS), 28034 Madrid, Spain; laura.lgonzalez@uah.es (L.L.-G.); raul.diazp@uah.es (R.D.-P.); 3 Department of Public and Maternal and Child Health, School of Medicine, Complutense University of Madrid, 28040 Madrid, Spain; jaleon@ucm.es (J.A.D.L.-L.); cbravoarribas@gmail.com (C.B.); prodriguezb@senefro.org (P.R.-B.); 4 Department of Obstetrics and Gynecology, University Hospital Gregorio Marañón, 28009 Madrid, Spain; 5 Health Research Institute Gregorio Marañón, 28009 Madrid, Spain; 6 Department of Nephrology, University Hospital Gregorio Marañón, 28009 Madrid, Spain; 7 Department of Surgery, Medical and Social Sciences, Faculty of Medicine and Health Sciences, University of Alcalá, 28801 Alcalá de Henares, Spain; 8 Immune System Diseases-Rheumatology and Internal Medicine Service, University Hospital Prince of Asturias, Networking Research Center on for Liver and Digestive Diseases (CIBEREHD), 28806 Alcalá de Henares, Spain; 9 Pathological Anatomy Service, University Hospital Gómez-Ulla, 28806 Alcalá de Henares, Spain

**Keywords:** late-onset preeclampsia (LO-PE), autophagy, placenta, ULK1, ATG9A, LC3, ATG5, STX-17, LAMP-1

## Abstract

Preeclampsia (PE) is a serious hypertensive disorder affecting 4–5% of pregnancies globally, leading to maternal and perinatal morbidity and mortality and reducing life expectancy in surviving women post-gestation. Late-onset PE (LO-PE) is a clinical type of PE diagnosed after 34 weeks of gestation, being less severe than the early-onset PE (EO-PE) variant, although both entities have a notable impact on the placenta. Despite the fact that most studies have focused on EO-PE, LO-PE does not deserve less attention since its prevalence is much higher and little is known about the role of the placenta in this pathology. Via RT-qPCR and immunohistochemistry methods, we measured the gene and protein expressions of several macroautophagy markers in the chorionic villi of placentas from women who underwent LO-PE (*n* = 68) and compared them to normal pregnancies (*n* = 43). We observed a markedly distinct expression pattern, noticing a significant drop in NUP62 expression and a considerable rise in the gene and protein expressions of ULK1, ATG9A, LC3, ATG5, STX-17, and LAMP-1 in the placentas of women with LO-PE. A major induction of autophagic processes was found in the placental tissue of patients with LO-PE. Abnormal signaling expression of these molecular patterns in this condition aids in the understanding of the complexity of pathophysiology and proposes biomarkers for the clinical management of these patients.

## 1. Introduction

Preeclampsia (PE) is a worrying hypertensive pregnancy disorder with a prevalence of 4–5% of pregnancies worldwide. PE is considered a leading cause of maternal and perinatal morbidity and mortality in severe cases [1], and decreases life expectancy in surviving women post-gestation. PE is diagnosed in pregnancy by new-onset hypertension (a systolic blood pressure ≥140 mm Hg or diastolic blood pressure ≥90 mm Hg on two occasions at least 4 h apart after 20 weeks of gestation), often accompanied by new-onset proteinuria [2]. Clinical management of this condition is quite limited, the “only cure” being delivery. However, some prophylactic measures, such as administration of a daily low dose of aspirin prior to 16 weeks of gestation, have been proven to prevent preterm PE in the early screening of mothers at risk [3] but not full-term PE [4]. Although its etiology is not well known, among the risk factors found are maternal hypertension, previous PE events, insulin-dependent diabetes, obesity, age (>40 years old), multiple pregnancies, and family history [5].

Depending on the time of appearance, there are two clinical types of PE: early-onset PE (EO-PE) or so-called placental PE (before 34 weeks of pregnancy) and late-onset PE (LO-PE) (after 34 weeks of pregnancy) [6]. EO-PE is intrinsically ligated to the placentation process; the pathophysiology proceeds with aberrant spiral artery remodeling that leads to hypoxia in the placenta, then local inflammation and oxidative stress provoke systemic inflammation in the mother with consequent aberrant endothelial and multiorgan functions. This kind of disorder is less common and more severe for the mother and fetus. On the contrary, the more prevalent LO-PE is also ligated to maternal extrinsic factors and shows vascular incompetence in placental molecular processes as well [7,8]. Nevertheless, the relationship between those intrinsic and extrinsic factors is not well elucidated yet, and little is known about the role of and changes occurring in the placental tissue in this condition. The key processes studied in both types of PE have been related to inflammation (vascular and systemic), angiogenesis incompetence, oxidative stress, or hypoxia [2]. The study of biomarkers to understand the differential chain links in these pathways may allow the management of these patients in clinical practice.

In previous studies, we found unbalanced immune responses in part due to the hyperactivation of inflammasomes such as NLRP3 and the release of proinflammatory cytokines [9]. Other authors found that closely connected processes like autophagy can negatively regulate the activation of inflammasomes, and those inflammatory mediators inhibit the functioning of autophagy [10]. In our current work, we emphasize the autophagy process to shed light on its contribution to the progression of LO-PE and reveal its interplay with inflammation and other hallmarks in the whole picture of this condition. The preserved cellular recycling process, known as autophagy, is essential for energy homeostasis and the adaptation of cells to any stress. It is a self-degradative process in which molecules, damaged organelles, and intracellular pathogens are cleaned to guarantee cell integrity and functions; thus, it is critical for cell proliferation, survival, gene stability, and senescence [11]. There are three primary forms of autophagy: macroautophagy (mediated by autophagosomes that absorb cytoplasmic components and join lysosomes), microautophagy (where materials are invaginated into lysosomes directly), and chaperone-mediated (where chaperones help in the selective transport of proteins to lysosomes) [12]. Macroautophagy is a process that occurs in different steps: initiation, nucleation, elongation, closure, maturation, fusion, and degradation [13]. The available literature has shown the reliability of different molecules in the study of macroautophagy, such as ULK1 and ATG9A (implicated in the initiation phase), LC3 and ATG5 (participating in elongation), STX-17 and LAMP-1 (involved in autophagosome and lysosome fusion), and NUP62/p62 (a negative regulator of the autophagic process) [14].

In the present study, we explore gene and protein expressions through RT-qPCR and immunohistochemistry of the aforementioned markers of macroautophagy in the placental tissue of a group of women with LO-PE (*n* = 68) and compare them with those obtained from normal pregnancies (*n* = 43).

## 2. Results

### 2.1. Clinical Characteristics of Patients

In this study, clinical characteristics of patients were meticulously examined, focusing on maternal age, nulliparous status, gestation duration, mode of delivery (C-section), and placental weight in both late-onset preeclampsia (LOPE) cases and healthy control pregnant women (HC). The mean maternal age for LOPE cases was 29 ± 4.8 years, showing a significant difference compared to HCs (31.4 ± 5.1 years, * *p* < 0.05). Notably, nulliparous women constituted a highly significant proportion of the LOPE group (77.9%) compared to HCs (32.6%) (*** *p* < 0.0001). The gestation period was slightly shorter in the LOPE group (38.6 ± 1.4 weeks) compared to HCs (39.1 ± 1.5 weeks). Additionally, the rate of C-section deliveries was comparable between the groups. Of particular significance was the difference in placental weight, where LOPE cases exhibited a significantly lower average weight (370.3 ± 61.7 g) compared to HCs (501 ± 65.3 g, *** *p* < 0.0001). These findings underscore the relevance of these clinical parameters in understanding and distinguishing cases of late-onset preeclampsia from normal pregnancies. The key clinical characteristics of the individuals under study are summarily presented in Table 1.

### 2.2. The Placentas of Women with Late-Onset Preeclampsia Exhibit Increased Expression of Autophagic Proteins Involved in the Initiation Phase, ULK-1 and ATG9A

**Figure 1:**
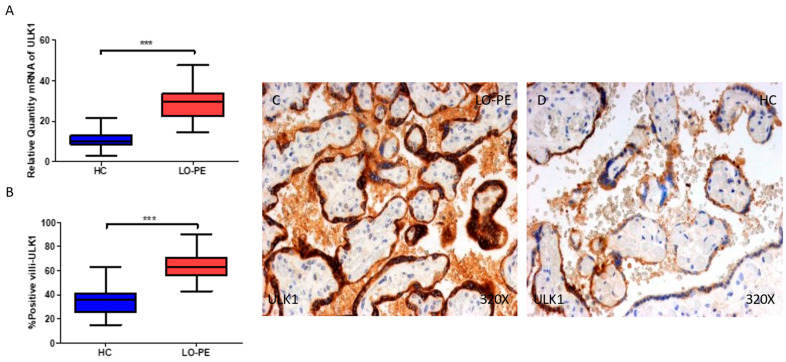
(**A**) mRNA expression of ULK-1 in women with LO-PE versus HCs. (**B**) IRS scores for ULK-1 in the chorionic villi of the LO-PE and HC groups. (**C**,**D**) Images showing immunostaining for ULK-1 in the chorionic villi of the LO-PE and HCs. *p* < 0.001 (***).

Firstly, we evaluated the gene and protein expressions of ULK-1 and ATG9A, two proteins implicated in the initiation phase in the placental tissue of women with LO-PE, and compared them with the HCs. Our results show that the gene expression of ULK-1 is notably higher in the placentas of women with LO-PE compared to HCs (*** *p* < 0.0001; LO-PE = 29.6 [14.6–47.6], HC = 10.2 [3–21.6], Figure 1A). The percentage of positive villi expressing ULK-1 defined by immunohistochemistry showed that the placental villi of women with LO-PE showed a notable upregulation in the expression of ULK-1, (*** *p* < 0.0001; LO-PE = 63 [43–90], HC = 36 [15–63], Figure 1B). Histological images comparing the pattern of expression of ULK-1 in the placentas of women with LO-PE versus HCs show that this protein is strongly expressed in the syncytiotrophoblast layer of women with LO-PE and in the inner cells of the chorionic villi, whereas for the HCs, the expression of ULK-1 is less marked and more limited to the syncytiotrophoblasts (Figure 1C,D).

**Figure 2:**
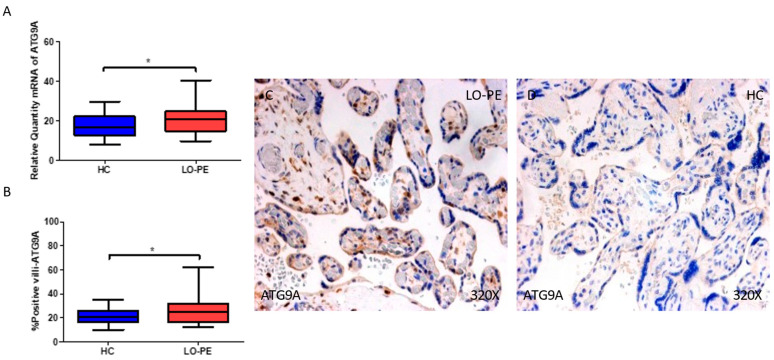
(**A**) mRNA expression of ATG9A in women with LO-PE versus HCs. (**B**) IRS scores for ATG9A in the chorionic villi of the LO-PE and HC groups. (**C**,**D**) Images showing immunostaining for ATG9A in the chorionic villi of the LO-PE and HCs. *p* < 0.05 (*).

On the other hand, we observed that the gene expression of ATG9A was also increased in the placentas of women with LO-PE when compared to HCs (* *p* = 0.013; LO-PE = 21.0 [9.8–40.3], HC = 16.6 [8.1–29.7], Figure 2A). The percentage of positive villi expressing ATG9A defined by immunohistochemistry showed that the placental villi of women with LO-PE showed a notable upregulation in the expression of this protein, (* *p* = 0.034; LO-PE = 25.0 [12.0–62.0], HC = 21.0 [10.0–35.0], Figure 2B). Histological images comparing the pattern of expression of ATG9A in the placentas of women with LO-PE versus HCs show that this protein is expressed in the syncytiotrophoblast layer and inner cells of the chorionic villi of women with LO-PE, whereas for the HCs, the expression of this protein is less marked and poorly observed (Figure 2C,D).

### 2.3. The Placental Tissue of Women with Late-Onset Preeclampsia Displays Augmented Expression of Autophagic Proteins Involved in the Elongation Phase, LC3 and ATG5

**Figure 3:**
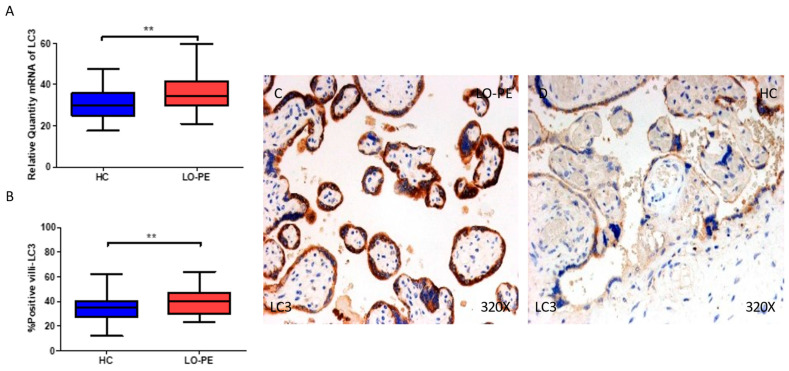
(**A**) mRNA expression of LC-3 in women with LO-PE versus HCs. (**B**) IRS scores for LC-3 in the chorionic villi of the LO-PE and HC groups. (**C**,**D**) Images showing immunostaining for LC-3 in the chorionic villi of the LO-PE and HCs. *p* < 0.01 (**).

We then assessed the gene and protein expressions of LC3 and ATG5, two proteins implicated in the elongation phase in the placental tissue of women with LO-PE, and compared them with HCs. Our results show that the gene expression of LC3 is notably upregulated in the placentas of women with LO-PE in comparison to HCs (** *p* = 0.002; LO-PE = 34.3 [21.0–59.8], HC = 30.1 [17.6–47.6], Figure 3A). The percentage of positive villi expressing LC3 defined by immunohistochemistry showed that the placental villi of women with LO-PE showed a notable upregulation in the expression of this protein (** *p* = 0.002; LO-PE = 40.0 [23.0–64.0], HC = 35.0 [12.0–62.0], Figure 3B). Histological images comparing the pattern of expression of LC3 in the placentas of women with LO-PE versus HCs show that this protein is strongly expressed in the syncytiotrophoblast layer of women with LO-PE, whereas for the HCs, the expression of LC3 is notably less marked (Figure 3C,D).

**Figure 4:**
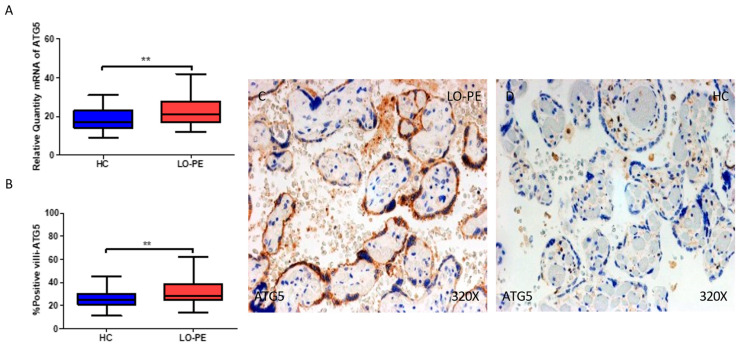
(**A**) mRNA expression of ATG-5 in women with LO-PE versus HCs. (**B**) IRS scores for ATG-5 in the chorionic villi of the LO-PE and HC groups. (**C**,**D**) Images showing immunostaining for ATG-5 in the chorionic villi of the LO-PE and HCs. *p* < 0.01 (**).

Regarding ATG5, we report that the gene expression of this protein is notably upregulated in the placentas of women with LO-PE in comparison to HCs (** *p* = 0.001; LO-PE = 21.1 [12–42], HC = 17 [9.0–31.1], Figure 4A). The percentage of positive villi expressing ATG5 defined by immunohistochemistry showed that the placental villi of women with LO-PE displayed a marked upregulation in the expression of this protein (** *p* = 0.002; LO-PE = 28.0 [14.0–62.0], HC = 25.0 [11.0–45.0], Figure 4B). Histological images comparing the pattern of expression of ATG5 in the placentas of women with LO-PE versus HCs show that this protein is strongly expressed in the syncytiotrophoblast layer of women with LO-PE, whereas for the HCs, the expression of ATG5 is more restricted to cells located in the inner chorionic villi (Figure 4C,D).

### 2.4. The Chorionic Villi of Women with Late-Onset Preeclampsia Show Augmented Expression of Autophagic Proteins Involved in the Fusion Phase, LAMP-1 and STX-17

**Figure 5:**
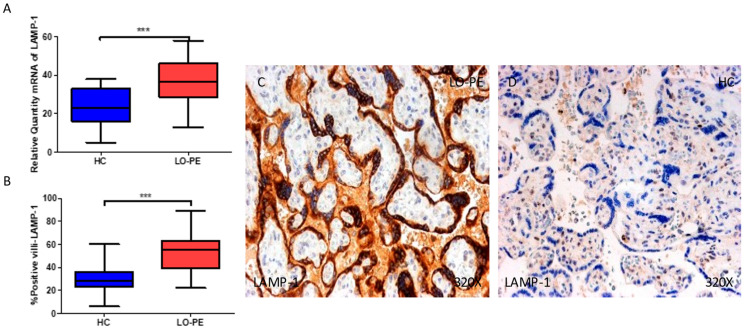
(**A**) mRNA expression of LAMP-1 in women with LO-PE versus HCs. (**B**) IRS scores for LAMP-1 in the chorionic villi of the LO-PE and HC groups. (**C**,**D**) Images showing immunostaining for LAMP-1 in the chorionic villi of the LO-PE and HCs. *p* < 0.001 (***).

We then assessed the gene and protein expressions of LC3 and ATG5, two proteins implicated in the elongation phase in the placental tissue of women with LO-PE, and compared them with the HCs. Our results show that the gene expression of LAMP-1 is notably higher in the placentas of women with LO-PE in comparison to HCs (*** *p* < 0.001; LO-PE = 36.5 [13–58], HC = 23.1 [5.0–37.9], Figure 5A). The percentage of positive villi expressing LAMP-1 defined by immunohistochemistry showed that the placental villi of women with LO-PE showed a notable upregulation in the expression of this protein (*** *p* < 0.001; LO-PE = 55.0 [22.0–89.0], HC = 28.0 [6.0–60.0], Figure 5B). Histological images comparing the pattern of expression of LC3 in the placentas of women with LO-PE versus HCs show that this protein is strongly expressed in the syncytiotrophoblast layer of women with LO-PE, whereas for the HCs, the expression of LC3 is less marked (Figure 5C,D).

**Figure 6:**
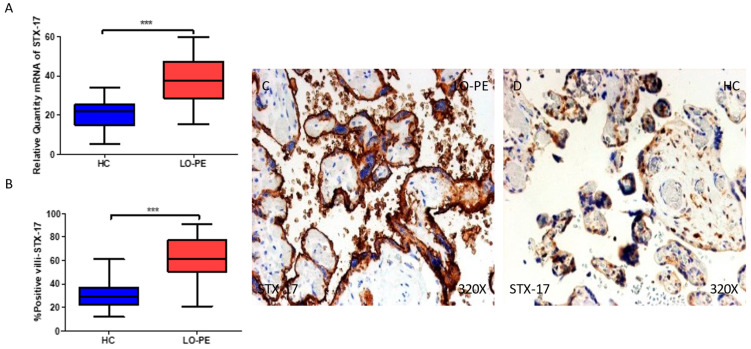
(**A**) mRNA expression of STX-17 in women with LO-PE versus HCs. (**B**) IRS scores for STX-17 in the chorionic villi of the LO-PE and HC groups. (**C**,**D**) Images showing immunostaining for STX-17 in the chorionic villi of the LO-PE and HCs. *p* < 0.001 (***).

Regarding STX-17, we report that the gene expression of this protein is remarkably upregulated in the placentas of women with LO-PE in comparison to HCs (*** *p* < 0.001; LO-PE = 37.3 [15.4–59.6], HC = 22.0 [5.3–34.0], Figure 6A). The percentage of positive villi expressing STX-17 defined by immunohistochemistry showed that the placental villi of women with LO-PE showed a notable upregulation in the expression of this protein (*** *p* < 0.001; LO-PE = 61.5 [21.0–91.0], HC = 29.0 [12.0–61.0], Figure 6B). Histological images comparing the pattern of expression of STX-17 in the placentas of women with LO-PE versus HC show that this protein is strongly expressed in the syncytiotrophoblast layer of women with LO-PE, whereas for the HCs, the expression of STX-17 is less marked (Figure 6C,D).

### 2.5. The Chorionic Villi of Women with Late-Onset Preeclampsia Report Diminished Expression of Autophagic Regulator NUP62

**Figure 7:**
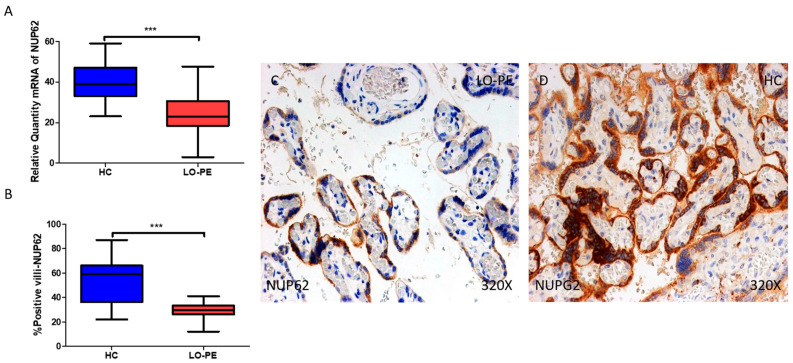
(**A**) mRNA expression of NUP62 in women with LO-PE versus HCs. (**B**) IRS scores for NUP62 in the chorionic villi of the LO-PE and HC groups. (**C**,**D**) Images showing immunostaining for NUP62 in the chorionic villi of the LO-PE and HCs. *p* < 0.001 (***).

We finally studied the gene and protein expressions of NUP62 in the chorionic villi of women with LO-PE and HCs. Regarding gene expression, there was a marked decrease in NUP62 in women with LO-PE (*** *p* < 0.001; LO-PE = 23.0 [3.0–47.6]; HC = 39.0 [23.1–59.0], Figure 7A). The percentage of positive villi expressing NUP62 defined by immunohistochemistry showed that the placental villi of women with LO-PE showed a notable downregulation in the expression of this protein (*** *p* < 0.001; LO-PE = 29.5 [12.0–41.0]; HC = 59.0 [22.0–87.0], Figure 7B). Histological images comparing the pattern of expression of LC3 in the placentas of women with LO-PE versus HCs show that this protein is strongly expressed in the syncytiotrophoblast layer and inner cells of the chorionic villi of the HC women, whereas for women with LO-PE, the expression of LC3 is less marked and more limited to syncytiotrophoblasts (Figure 7C,D).

## 3. Discussion

LO-PE is an obstetric disorder that shows specific molecular patterns in placental tissue. The higher metabolic demands of the fetoplacental unit are thought to cause mechanical constraints and intraplacental (intervillous) malperfusion, which is thought to be the secondary cause of LO-PE [15]. Different studies have tried to explain these hypotheses by studying oxidative stress, endothelial impairment, modified mitochondrial function, and apoptosis and inflammation [16,17]. To shed more light on the comprehension of this pathology, we focused on autophagy processes, which are closely connected to the abnormal environment associated with PE.

Autophagy is an essential process active at basal levels but that acquires a more important role as an adaptative survival process in response to nutrient deprivation and different intracellular and extracellular stressors [18]. This process can be either selective or non-selective in the removal of specific organelles, ribosomes, and protein aggregates, although the regulatory aspects of selective autophagy are not fully understood [11]. An altered regulation of the autophagic process has been linked to different human diseases [18]. In the placenta, this process is essential in early pregnancy, and different obstetric conditions like PE or fetal growth restriction (FGR) seem to be associated with an abnormally increased placental autophagosome formation, mainly related to different pathological factors like hypoxia, endoplasmic reticulum stress, altered mTOR activity, exacerbated inflammation, and abnormal functioning of trophoblasts [19]. The relationship between the autophagic process and LO-PE, however, remains an area of active investigation, and the precise role of autophagy in the placental tissue of women affected by this condition needs to be fully covered [20].

In our study, we observed that certain signaling elements of macroautophagy show differential expression in the placenta of LO-PE patients, such as concretely exacerbated increases in ULK1, ATG9A, LC3, ATG5, STX-17, and LAMP-1 and a significant decrease in NUP62. These findings suggest that the autophagy process might be playing a crucial role in the pathogenesis of LO-PE, although further studies are warranted to confirm our results.

Firstly, we reported an augmented expression of ULK1 and ATG9A, two markers of autophagy implicated in the initiation step [21]. To our best knowledge, an augmented or altered expression of ULK1 and ATG9A in the placental tissue has not yet been linked to LO-PE, but some studies relating autophagy and pregnancy in animal and non-animal models may help in the understanding of the differential expressions of these markers observed in our study. An increased expression of ULK1 has been observed in the placental tissue of women with gestational diabetes mellitus (GDM) when compared to healthy women [22]. Similarly, a bioinformatic analysis identified ULK1 as one of the 250 candidate genes upregulated in the placentas of women with PE [23], a finding that is supported by our study. Regarding ATG9A, animal models have found that both hetero- and homozygous deletions of this protein are associated with the development of PE and FGR [24]. On the other hand, reduced expression of this protein has been observed after hepatitis E virus infection [25]. Collectively, these studies suggest the potential pathogenic role of ULK1 and ATG9A in the placentas of women with LO-PE, although further efforts are warranted to evaluate possible translational implications derived from these molecules.

Elongation factors LC3 and ATG5 have also been shown to be increased in the chorionic villi of LO-PE women in our study. Yet, previous studies have found increased autophagic activity through increased expression of LC3 proteins in human placental trophoblasts of severe PE (but not distinguishing between EO or LO-PE) [26]. Conversely, other studies using immunohistochemistry techniques as well found reduced LC3-II expression despite autophagy deficiency [27]. In other research, more focused on epigenetic changes, it was found that the silencing of certain regulators like histone deacetylase 4 (HDAC4) could upregulate the activation of autophagy components, including LC3, and apoptosis in placental trophoblast cells in general PE [28]. We did not find in the literature experimental data for ATG5 evidence in PE models. Some in vitro studies in GDM have tried to ameliorate augmented autophagy and apoptosis through the knockdown of ATG5 with success [29].

Furthermore, a recent study found changes in the molecular expression of some of these markers, among others, in the peripheral blood of preeclamptic women, and, in fact, revealed that their expressions were not only higher in PE women compared to HCs but were even higher in LO-PE cases than in EO-PE ones. Concretely, they found higher expressions of ATG5 and LC3B through Western blotting assays, concluding there is high autophagy activation in these patients [30].

Next, LAMP1 (lysosomal-associated membrane protein 1) and STX-17 (syntaxin 17), involved in fusion steps, were also found to be increased in the chorionic villi of LO-PE women. In serum and placental samples from severe PE cases, cell lines were treated to complete assays of immunofluorescence, animal models, and Western blotting with the aim of studying autophagy routes in normotensive and preeclamptic conditions. In vitro findings suggested that LAMP1 was reduced in autophagy-deficient cells [31]. Conversely, similar techniques in different studies using serum and placental tissue found decreased LAMP1 levels in trophoblasts associated with lysosomal dysfunction and autophagy inhibition [32]. Moreover, STX17, a less-characterized protein in these processes, is known to be anchored to smooth endoplasmic reticulum and abundantly expressed in steroidogenic cells, being necessary to synthesize progesterone to assure the relation between the mother and the fetus [33,34]. Further research is still required for relating this protein with autophagy processes, especially in pregnancy and more concretely in PE, EO-PE, and LO-PE patients.

Lastly, the nuclear pore glycoprotein p62, sequestosome 1 (or NUP62) is an autophagosome cargo protein which binds to LC3 and other autophagy elements to present ubiquitinated cytoplasmic proteins to autophagosomes for degradation in lysosomes. Decreased levels of NUP62 are observed when autophagy is induced, this marker being a good indicator for autophagy flux [35]. NUP62 has previously been evidenced to be accumulated in preterm villous placentas, associated with a downregulation of the autophagy route. This impairment was also found to be related to a lower birth weight, suggesting that autophagy homeostasis is key for fetal growth [36]. Recent studies have found different results in LO-PE patients, where mRNA levels of this protein were less expressed but protein expression was higher, as determined through enzyme-linked immunosorbent assay (ELISA) techniques [37]. Another Western blotting analysis in placental tissue also found a significantly reduced expression of NUP62 with increased LC3 in general PE, but remarked that these changes were noted in the presence or absence of FGR, therefore indicating that autophagy is active in hypertensive obstetric disorders even without the manifestation of FGR [38].

In summary, our findings suggest a discernible upregulation of autophagy in the context of LO-PE. To elucidate the intricate connections between these pathways and others integral to the pathophysiology of this condition, future investigations of greater depth are warranted. A notable limitation in our current study pertains to the constrained availability of clinical data concerning both maternal and fetal aspects, which, if expanded, would facilitate more robust statistical correlations. The incorporation of additional clinical data is imperative for the translational application and relevance of our molecular results.

Moreover, our study is confronted with the inherent drawback of lacking data related to EO-PE, thus precluding a comparative analysis of autophagic markers between both subtypes of PE. Obtaining data for EO-PE presented considerable challenges attributed to its heightened severity, coupled with its lower prevalence. To our best knowledge, the scientific literature about these metabolic processes of autophagy with the distinction of both subtypes of the disease is limited. Subsequent research endeavors should be directed toward overcoming these limitations, necessitating the acquisition of more comprehensive clinical data and a comparative exploration of autophagic markers in both LO-PE and EO-PE.

## 4. Materials and Methods

### 4.1. Study Design and Participants

A prospective, observational study was conducted and nested within a cohort for comparative analysis. Patients with preeclampsia who satisfied some of the following severity criteria were diagnosed with LO-PE following the American College of Obstetricians and Gynecologists Practice Guidelines for Gestational Hypertension and Preeclampsia [39]: systolic (≥160 mmHg) and/or diastolic (≥110 mmHg) blood pressure measurements confirmed after 15 min; urine protein/creatinine ratio estimated or measured in 24 h; oliguria ≤500 mL/24 h or diuresis rate <0.5 mL/kg/h for two hours; renal failure, defined as serum creatinine > 1.1 mg/dL or twice the serum creatinine value in the absence of other renal disease; hematological disorders, such as thrombocytopenia (<100,000 mm^3^), disseminated intravascular coagulation (DIC), or hemolysis; neurological or visual disturbances, such as severe headache that does not go away with analgesics, blurred vision, diplopia, or amaurosis; acute pulmonary edema or cyanosis; pain in the epigastrium or right hypochondrium; liver dysfunction, defined as transaminase levels elevated to twice the normal value; and placental involvement with fetal symptoms such as fetal mortality, aberrant umbilical artery Doppler readings, and intrauterine growth restriction (IGR) [40,41]. In this investigation, the criterion for the degree of preeclampsia severity was defined as the existence of a blood creatinine level greater than 1.1 mg/dL [39]. In addition, 43 pregnant women who were free of ailments were classified as healthy controls (HCs) and were included in the study.

### 4.2. Sample Processing

Placental biopsies were taken following delivery. In every instance, the placenta was cut into five sections to guarantee that the sample included a range of cotyledons. Then, these fragments were placed in a sterile tube that contained 1% antibiotic/antimycotic and Minimum Essential Medium (MEM) from ThermoFisher Scientific, Waltham, MA, USA. All samples were refrigerated and delivered to the laboratory within two hours. A laminar class II laminar flow hood (Telstar AV 30/70 Müller 220 V 50 MHz; Telstar SA Group, Terrassa, Spain) was used to process the samples in a sterile setting. Subsequently, the MEM samples underwent immunodetection and histopathology analyses.

Placental fragments kept in the MEM were divided into pieces using conventional procedures, and blood cells were then extracted by fixing the pieces in F13 (60% ethanol, 20% methanol, 7% polyethylene glycol, and 13% distilled water). Molds were used to originally incorporate paraffin blocks. After the paraffin had set, 5 µm thick slices were cut using an HM 350 S rotation microtome (Thermo Fisher Scientific, Waltham, MA, USA). After that, the sections were placed in a hot water bath and gathered onto a glass slide that had been coated with 10% polylysine beforehand to improve the adhesion of the incisions.

### 4.3. Gene Expression Determination

The target genes’ expressions were examined using quantitative reverse transcription polymerase chain reaction (RT-qPCR). The concentration of cDNA (Thermo Fisher Scientific) in every sample was determined. The RNA was extracted using the guanidine–phenol–chloroform isothiocyanate method [42], and the primers that were utilized were created using the Auto-Dimer program and the Primer-BLAST tool [43,44]. Using the StepOnePlusTM apparatus and the relative standard curve method, we carried out qPCR.

After being diluted with nuclease-free water, 5 µL of each sample was mixed with 10 µL of the intercalating agent iQTM SYBR^®^ Green Supermix (Bio-Rad Laboratories, Hercules, CA, USA), 1 µL of each primer (forward and reverse), and 3 µL of well plate DNase- and RNase-free water. The 20 µL solutions were assessed using a MicroAmp^®^ 96-(Applied Biosystems-Life Technologies, Foster City, CA, USA). To normalize and compare the final data, a housekeeping gene called glyceraldehyde 3-phosphate dehydrogenase (GAPDH; Table 2) was utilized. The data gathered for every gene were interpolated using the standard curve. Two tests were conducted on the standard curve, three tests were conducted on the samples, and the remaining two wells were filled with negative controls.

### 4.4. Immunohistochemistry

The ABC (avidin–biotin complex) method was used to explore the detection of an antigen–antibody response, with peroxidase serving as the chromogen, in accordance with the known protocols [45,46]. The primary antibody incubation (Table 3) was conducted overnight at 4 °C and was performed using a 3% BSA and PBS dilution from Abcam (Cambridge, UK). Conversely, the secondary antibody that was diluted in PBS and coupled to biotin was incubated for 1.5 h at room temperature.

The chromogenic substrate diaminobenzidine (Kit DAB, SK-4100, Vector Laboratories, Burlingame, CA, USA) was used for 60 min at room temperature (in a PBS 1:200 dilution) after being prepared shortly before exposure (5 mL of distilled water, two drops of buffer, four drops of DAB, and two drops of hydrogen peroxide). Within this procedure, brown staining is conceivable. In every immunohistochemistry experiment, sections from the same tissue were used as the negative controls. In these experiments, the primary antibody incubation was replaced by an incubation in PBS, a blocking solution.

To count the immunopositive cells in tissue slices, five counts were randomly applied, excluding any cells that did not pass the predetermined demarcation lines. As per the anatomopathological criteria delineated by prior research, patients were deemed positive if the immunoreactive score (ISR score) for each subject exceeded or was equal to 5% of the overall test sample score [47]. An optical microscope, a Carl Zeiss Axiophot, was used to analyze the cuts. The tissue’s immunostaining was assessed by two different histologists, who were blinded to the outcome measure.

### 4.5. Statistical Analysis

The statistical analysis was conducted using GraphPad Prism^®^ 6.0, and a Whitney U test was run. Data are expressed using the median and the interquartile range (IQR). The significance was assessed using *p* 0.05 (*), *p* 0.01 (**), and *p* 0.001 (***) values.

## 5. Conclusions

**Figure 8:**
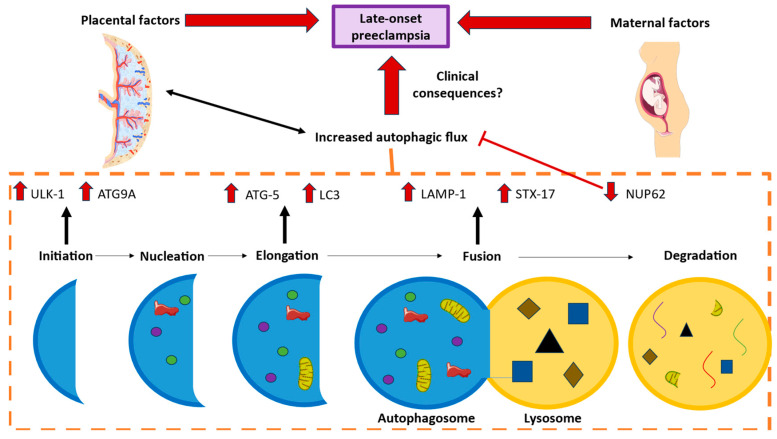
Graphical abstract illustrating the observed results in the current study. In this investigation, we meticulously selected various protein markers intricately involved in distinct stages of macroautophagy. Utilizing immunohistochemical techniques, we sought to gain a comprehensive understanding of the pathophysiological processes underlying late-onset preeclampsia (LO-PE). Comparative analyses between patients afflicted with this condition and those experiencing uncomplicated pregnancies revealed heightened expression levels of ULK-1 and ATG9A (implicated in the initiation phase), ATG-5 and LC3 (associated with the elongation phase), as well as LAMP-1 and STX-17 (pertaining to the fusion of autophagosome and lysosome). Concurrently, a diminished expression of NUP62 (a negative regulator) was observed. These alterations are believed to stem from a combination of maternal and placental (or intrinsic) factors characteristic of the LO-PE process. It is imperative to underscore that future research endeavors are necessary to elucidate the potential clinical consequences resulting from the imbalance in the expression of these identified markers.

In the present comparative study, we explored a significantly differential expression pattern of the macroautophagy process in the chorionic villi of placentas from women who suffered from LO-PE. We observed marked increased gene and protein expressions of ULK1, ATG9A, LC3, ATG5, STX-17, and LAMP-1 and a notably decreased expression of NUP62, explaining the major induction of autophagic processes, as summarized in Figure 8. Knowledge on this abnormal signaling expression contributes to the understanding of the intricate pathophysiology of LO-PE and proposes biomarkers of analysis for the clinical management of these patients.

## Figures and Tables

**Table 1:** Clinical features of the subjects included in our study. * = *p* < 0.05; *** = *p* < 0.001.

	HC (*n* = 43)	LO-PE (*n* = 68)	*p*-Value
Maternal age (years) mean ± SD	31.4 ± 5.1	29 ± 4.8	* *p* = 0.0154
Nulliparous (%) total number	14 (32.6)	53 (77.9)	*** *p* < 0.0001
Gestation (weeks)	39.1 ± 1.5	38.6 ± 1.4	NS (*p* = 0.075)
C-section (%) Total number	8 (18.6)	15 (22.1)	NS (*p* = 0.270)
Placental weight (g)	501 ± 65.3	370.3 ± 61.7	*** *p* < 0.0001

**Table 2:** Primers selected for each gene.

GENE	SEQUENCE Fwd (5′ → 3′)	SEQUENCE Rev (5′ → 3′)	Temp
TBP	TGCACAGGAGCCAAGAGTGAA	CACATCACAGCTCCCCACCA	60 °C
ULK1	GTTCCAAACACCTCGGTCCT	CGATCTCCATGGGCTTCTCC	59 °C
LC3	GAGTTACCTCCCGCAGCC	ACCCAGAGGGACAACCCTAA	60 °C
NUP62	CGAGGTGGATGTCCGTCTTT	GTCTGCAGCCTTGGGAAGAT	61 °C
STX17	CCCGGCGGGAGGTTTTT	AAGTCAGTGACCAGTTGGCT	60 °C
LAMP1	GGCCTCTTGCGTCTGGTAAC	AAAGGTACGCCTGGATGGTG	57 °C
ATG9A	GGCTGGAGAGGAGCACATAC	ACCAGCAATGACCAGGATGG	60 °C
ATG5	GCAACTCTGGATGGGATTGC	TTGCAGCAGCGAAGTGTTTC	61 °C

**Table 3:** Primary and secondary antibodies used in our study and their dilutions.

Antigen	Species	Dilution	Provider	Protocol Specifications
ULK1	Rabbit polyclonal	1:100	Abcam (ab203207)	10 mM sodium citrate. pH = 6, before incubation with blocking solution
LC3	Rabbit monoclonal	1:150	Abcam (ab192890)	------
NUP62	Rabbit monoclonal	1:1000	Abcam (ab207305)	EDTA, pH = 9, before incubation with blocking solution
STX17	Rabbit polyclonal	1:200	Abcam (ab229646)	------
LAMP1	Rabbit polyclonal	1:250	Abcam (ab24170)	EDTA, pH = 9, before incubation with blocking solution
ATG9A	Rabbit monoclonal	1:500	Abcam (ab108338)	100% Triton 0.1% in PBS for 10 min before incubation with blocking solution
ATG5	Rabbit monoclonal	1:100	sc-133158	100% Triton 0.1% in PBS for 10 min before incubation with blocking solution
IgG (Rabbit)	Mouse	1:1000	Sigma-Aldrich (Saint Louis, MO, USA) (RG96/B5283)	------

## Data Availability

The data used to support the findings of the present study are available from the corresponding author upon request.
